# DNA Barcoding, GIS-Facilitated Seed Germination and Pilot Cultivation of *Teucrium luteum* subsp. *gabesianum* (Lamiaceae), a Tunisian Local Endemic with Potential Medicinal and Ornamental Value

**DOI:** 10.3390/biology11030462

**Published:** 2022-03-17

**Authors:** Stefanos Kostas, Stefanos Hatzilazarou, Elias Pipinis, Soumaya Bourgou, Imtinen Ben Haj Jilani, Wafa Ben Othman, Wided Megdiche-Ksouri, Zeineb Ghrabi-Gammar, Mohamed Libiad, Abdelmajid Khabbach, Mohamed El Haissoufi, Fatima Lamchouri, Emmanouil Koundourakis, Vasileios Greveniotis, Evgenia Papaioannou, Michalia A. Sakellariou, Ioannis Anestis, Georgios Tsoktouridis, Nikos Krigas

**Affiliations:** 1 Laboratory of Floriculture, School of Agriculture, Aristotle University of Thessaloniki, 54124 Thessaloniki, Greece; skostas@agro.auth.gr (S.K.); hatzilaz@agro.auth.gr (S.H.); ekoundou@agro.auth.gr (E.K.); 2 Laboratory of Silviculture, School of Forestry and Natural Environment, Aristotle University of Thessaloniki, 54124 Thessaloniki, Greece; epipinis@for.auth.gr; 3 Laboratoire des Plantes Aromatiques et Médicinales, Centre de Biotechnologie de Borj-Cédria, Tunis 2050, Tunisia; ksouriwided@yahoo.fr; 4 Institut National Agronomique de Tunisie, Université de Carthage, Tunis 1082, Tunisia; imtinenbhj@yahoo.fr (I.B.H.J.); wafabenothman@gmail.com (W.B.O.); zghrabi@yahoo.fr (Z.G.-G.); 5 Laboratoire de Recherche Biogéographie, Climatologie Appliquée et Dynamiques Environnementales (BiCADE 18ES13), Faculté des Lettres des Arts et des Humanités de Manouba, Campus Universitaire de la Manouba, Université de la Manouba, Manouba 2010, Tunisia; 6 Laboratory of Natural Substances, Pharmacology, Environment, Modelling, Health and Quality of Life (SNAMOPEQ), Polydisciplinary Faculty of Taza, Sidi Mohamed Ben Abdellah University, Taza 35000, Morocco; libiad001@gmail.com (M.L.); khamajid@hotmail.com (A.K.); mohamed.elhaissoufi1@usmba.ac.ma (M.E.H.); fatima.lamchouri@usmba.ac.ma (F.L.); 7 Laboratory of Ecology, Systematics and Biodiversity Conservation (LESCB), URL-CNRST Nº18, FS, Abdelmalek Essaadi University, Tetouan 93000, Morocco; 8 Laboratory of Biotechnology, Conservation and Valorization of Natural Resources (BCVRN), Department of Biology, Faculty of Sciences Dhar El Mahraz, Sidi Mohamed Ben Abdellah University, Fes 30003, Morocco; 9 Institute of Industrial and Forage Crops, Hellenic Agricultural Organization Demeter, 41335 Larisa, Greece; vgreveni@mail.com; 10 Laboratory of Forest Soil Science, School of Forestry and Natural Environment, Aristotle University of Thessaloniki, 54124 Thessaloniki, Greece; eapapaioa@agro.auth.gr; 11 Institute of Plant Breeding and Genetic Resources, Hellenic Agricultural Organization Demeter, 57001 Thessaloniki, Greece; michsakellariou@yahoo.com (M.A.S.); ganestis3@gmail.com (I.A.); gtsok1@yahoo.co.uk (G.T.); 12 Theofrastos Fertilizers, Industrial Area of Korinthos, Irinis & Filias (Arion), 20100 Korinthos, Greece

**Keywords:** African biodiversity, integrated nutrient management, photosynthetic rate, nutrient content, sustainable exploitation, neglected and underutilized plants, in situ and ex situ conservation

## Abstract

**Simple Summary:**

Neglected and underutilized plants (NUPs) are significant resources that are often endemic to small geographical territories. Focusing on the Tunisian local endemic *Teucrium luteum* subsp. *gabesianum* (Lamiaceae) and using Geographical Information Systems and online databases, we created a detailed ecological profiling for this taxon in terms of temperature and climate conditions required for sustaining wild-growing populations in situ, and we showed how temperature affects its ex-situ seed germination, thus making its cultivation in anthropogenic environments possible for the first time. We further investigated the growth and pilot cultivation of seedlings responding to chemical and integrated nutrient management (INM) fertilizers, outlining the advantageous effect of INM. The first-reported herein DNA barcoding offers insight regarding the taxonomic circumscription of other *Teucrium* taxa, enables future traceability in products, and may be useful in future product design. The multidisciplinary approach followed herein may bridge research gaps hindering the conservation efforts and/or the sustainable exploitation of this local endemic NUP of Tunisia to date. As a result, the feasibility and readiness timescale for its sustainable exploitation was re-evaluated, upgrading (>two-fold) its potential value for the medicinal-cosmetic, agro-alimentary, and ornamental-horticultural sectors.

**Abstract:**

In the context of plant conservation and sustainable use of unique neglected and underutilized phytogenetic resources, this study focused on the Tunisian local endemic *Teucrium luteum* subsp. *gabesianum* (Lamiaceae). Using Geographical Information Systems and online databases, detailed taxon-specific ecological profiling was produced for the first time, which illustrated the temperature and climate conditions in its wild habitats and facilitated the investigation of how temperature affects its seed germination, thus making its cultivation in anthropogenic environments possible. Following the seed propagation first reported herein (77.5–81.25% at temperatures between 15 and 25 °C), species-specific in situ and ex situ conservation efforts or sustainable exploitation strategies can be enabled. This study also reported for the first time how chemical and integrated nutrient management (INM) fertilizers affect the growth and pilot cultivation of its seedlings (INM more advantageous). The firstly-reported herein DNA barcoding may enable its traceability, allowing future product design. The multidisciplinary approach followed has paved the way to bridge important research gaps hindering conservation efforts and/or the sustainable exploitation of this local Tunisian endemic plant to date. Based on the aforementioned results, the feasibility and readiness timescale for its sustainable exploitation was overviewed and re-evaluated herein, upgrading (>two-fold) its potential value for the medicinal-cosmetic, agro-alimentary, and ornamental-horticultural sectors.

## 1. Introduction

The genus *Teucrium* (Lamiaceae) consists of 286 taxonomically validated species and subspecies (https://powo.science.kew.org/, accessed on 13 December 2021), 23 of which are included in the Tunisian flora [1]. These species are mainly perennial plants (rarely annual or biennial) of dry, arid, and rocky places with open vegetation, and most of them are rich in bioactive compounds. Several members of this genus are an abundant source of neo-clerodane diterpenoids and phenolics [2,3].

*Teucrium* species have been used for more than 2000 years as medicinal plants, and they are known for their important properties [4]. In North Africa, *Teucrium* species are commonly used as hypoglycemic and healing agents [3]. Species of genus *Teucrium* have been used in ethnopharmacology for centuries to treat many ailments, diseases of the digestive system, cold, flu, and fever, as stimulants, antiseptic, astringent, tonic diaphoretic, and in cicatrizing remedies, as well as in diabetes and tuberculosis treatments [4]. The decoctions and infusions represent the most common preparation for plants of this genus [5]. *Teucrium polium* L. has been used traditionally as a diuretic, antipyretic, antispasmodic, and coagolic [6]. In traditional African medicine, this species is used for its antistress property, allowing relaxation, promoting sleep, and helping to stimulate memory, for headaches and gastrointestinal disorders, as well as analgesic, anorexic, antipyretic, cholagogue, febrifuge, tonic, dewormer, and antispasmodic effects [7]. In Tunisia, various closely related taxa of *Teucrium* are locally called without discrimination “Al-Ja’adeh”, “Khayata”, and “Gattaba”, which means “cicatrisant” (especially taxa of the section *Polium*), a name which reflects widespread traditional use. These vernacular names referring to different taxa of section *Polium* are mentioned in the traditional Tunisian pharmacopoeia because of their medicinal properties particularly the healing of wounds [8,9,10,11].

*Teucrium* plants are not commonly cultivated and are usually sourced directly from the wild. The uncontrolled and intensive harvesting of medicinal and aromatic plants (MAPs) from their natural environment poses great pressure on the wild populations of MAP species, threatening their existence, and consequently leading to a significant depletion of genetic resources [12,13,14]. Therefore, it is highly important to consider the conservation of these species, focusing on range-restricted and local endemic plants. The development of propagation and cultivation protocols for rare and/or endemic species could facilitate conservation and restoration efforts. Seed propagation is followed for the majority of species in conservation efforts to maintain genetic diversity but also for sustainable exploitation, since it is a rather easy and low-cost method, typically applied in nurseries [15].

*Teucrium luteum* (Mill.) *Degen* subsp. *gabesianum* (S. Peutch) Greuter & Burdet is a wild-growing local endemic subspecies of Tunisia [16] that is closely related to one of the most commonly known aromatic medicinal plants in this genus, *T. polium* [17]. To date, phytochemichal and biological investigations have paid no attention to this taxon, and there are no references regarding its chemical composition and biological effects [18]. Despite its promising potential in the agro-alimentary sector [19], the ornamental-horticultural sector [20], and the medicinal-cosmetic sector [18], many research gaps still exist for this valuable but neglected and underutilized plant (NUP). The seed ecology of *T. luteum* subsp. *gabesianum* has not been investigated yet, and therefore, there is a great lack of information regarding its ecology and germination requirements [20]. Understanding the germination process and requirements of *T. luteum* subsp. *gabesianum* in its natural habitat, as well as the environmental factors affecting them will provide useful insight for its in-situ and ex-situ conservation. Seed germination studies facilitated with Geographic Information Systems (GIS) may be used to guide seed germination trials and seed storage and to achieve optimal seed germination in conservation efforts and sustainable exploitation strategies [21,22,23].

Moreover, *T. luteum* subsp. *gabesianum* has never been cultivated ex-situ [16], and information regarding seedling production of *T. luteum* subsp. *gabesianum* in pots or containers is also missing, thus compromising further usability and applied research. In general, the production of high-quality planting stock remains the main challenge for nurseries in sustainable exploitation efforts and at the same time it is a critical factor for the successful cultivation and ex situ conservation of rare species in harsh Mediterranean environmental conditions (high temperatures, limited water resources, and low soil fertility) [24]. Plant morphological and physiological characteristics can be improved through nursery cultivation practices, providing a high-quality starting material for further field cultivation [25]. The morphological and physiological attributes of planting stock define the quality of seedlings [26], including the use of inorganic and organic fertilizers, as well as integrated nutrient management regimes. Since the restoration programs focused on rare and endemic plants often take place on poor and degraded soils, especially in Mediterranean areas, it is important to recognize how to increase the nutrient reserves of plants at the nursery stage [27]. To this end, it has been reported that the application of nitrogen fertilizers in nursery conditions usually increases the survival and growth of plant seedlings both at the nursery and in the field [28,29].

Considering the importance of local endemic species conservation and the potential of *T. luteum* subsp. *gabesianum* to be introduced as a new cultivated NUP, focus should be given to the study of the abiotic conditions in which the species is naturally adapted as a proxy of species-specific tolerance in terms of temperature and climatic conditions. To this end, GIS-derived species-specific ecological profiles [21,22] can generate the necessary information concerning the range of abiotic factors prevailing in its wild habitats and thus can facilitate seed germination and pilot ex-situ cultivation [23,30].

The assessment of genetic diversity in endemic species is another key factor in conservation strategy planning and genetic resource management. The genus *Teucrium* with more than 286 species and subspecies grouped in nine sections is a valuable phytogenetic resource that remains unexploited at a great level, as NUPs [3]. Molecular markers are a powerful tool for the evaluation of the genetic variability in several species. RAPD molecular markers have been used in genetic studies of six *Teucrium arduini* L. populations [31], as well as among and between *T. polium* populations [32,33]. Genetic diversity was estimated using ISSR molecular markers in *T. polium* populations [34,35] and in the local endemic species of Turkey *Teucrium leucophyllum* Montbret & Auscher ex Benth [36], while [37] used both RAPD and ISSR molecular markers for the molecular characterization of eleven *Teucrium* species. Genetic variability was assessed in *T. arduini* by AFLP analysis [38]. DNA barcoding based on chloroplast DNA molecular markers is a promising method or technique for species and/or varieties’ identification, generating a taxon-specific fingerprint that allows traceability. Furthermore, to estimate the phylogenetic relationships among members of *Teucrium*, a phylogenetic study has been performed using nuclear ribosomal (ITS region) and chloroplast DNA (*ndh*F gene, *trn*L-F region) for *Teucrium flavum* L., *T. scorodonia* L., and *T. polium* [39,40] and DNA sequence data from 101 *Teucrium* taxa [41]. Moreover, sequencing of the whole chloroplast DNA genome has been completed only for six *Teucrium* species, i.e., *T. stocksianum* Boiss. subsp. *stocksianum*, *T. stocksianum* Boiss. subsp. *stenophyllum* R.A. King, *T. mascatnese* Boiss. [42], *T. ornatum* Hemsl (MN814864), *T. simplex* Vaniot (MN814872), and *T. omeiense* Y. Z. Sumn (MN814871), providing valuable information for the genetic classification of *Teucrium* taxa.

In this context, the primary aims of the present study were to: (i) Provide genetic information about *T. luteum* subsp. *gabesianum* in GenBank as a reference for comparison to other *Teucrium* species; (ii) Define the ecological requirements of the focal taxon in its wild habitats with the use of GIS; (iii) Investigate its germination requirements in the lab and more precisely to examine the effect of temperature on seed germination; (iv) Assess the effect of inorganic and integrated nutrient management fertilizers on seedling growth by means of seedling morphological and physiological properties; and (v) Compare the effect of fertilizers on seedlings in terms of nutrient content. Since *T. luteum* subsp. *gabesianum* is only confined to the northern parts of Tunisia, the aforementioned were envisaged herein as contributions to the conservation and sustainable use of unique MAPs and NUPs [18,19,20,43]. Consequently, the new data produced in this study may actually bridge extant applied research gaps, thus permitting the re-evaluation of *T. luteum* subsp. *gabesianum* in terms of feasibility for value chain creation and readiness timescale for sustainable exploitation in the future [20,44].

## 2. Materials and Methods

### 2.1. Multifaceted Evaluation in Different Sectors of Economy

The calculation of the potential of *T. luteum* subsp. *gabesianum* in specific economic sectors (Level I evaluation) was based on (i) twenty specific attributes assessing its ornamental-horticultural value [20], (ii) seven attributes assessing its agro-alimentary value [19], and (iii) nine attributes assessing its value for the medicinal-cosmetic sector [18]. This new methodological scheme is described in detail per economic sector with guidelines and examples of scorings for 399 local endemic taxa of three Mediterranean regions (Crete, Mediterranean Coast–Rif, and Tunisia) in previous studies [18,19,20]. All scoring of individual attributes per economic sector were expressed as relative percentage (%) of the maximum possible score that could be generated [18,19,20].

The evaluation of feasibility for value chain creation regarding *T. luteum* subsp. *gabesianum* (Level II evaluation) involved point-scoring of 12 selected attributes see details in [20]. The nominated readiness timescale assessment for the sustainable exploitation of *T. luteum* subsp. *gabesianum* (Level III evaluation) was based on previous SWOT (Strengths, Weaknesses, Opportunities, Threats) and gap analyses [20]. Although these evaluations were sourced from previous studies [18,19,20], the data presented herein for *T. luteum* subsp. *gabesianum* are presented in detail for the first time.

### 2.2. Distribution Mapping and GIS Ecological Profiling

**Figure 1:**
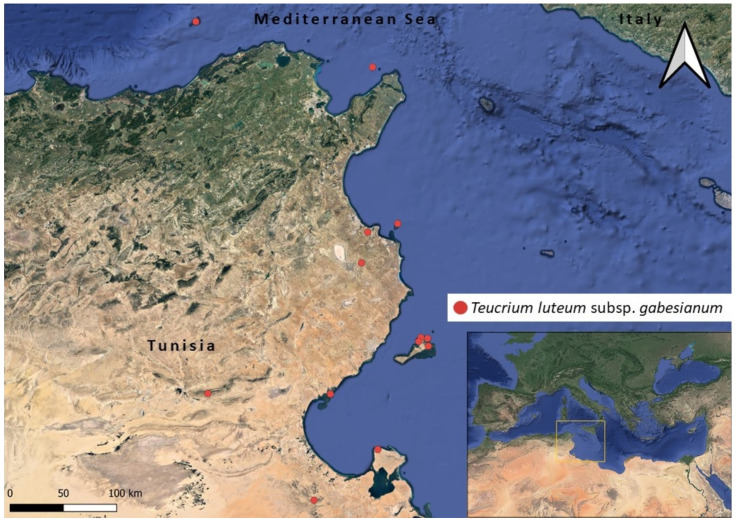
Overview of the known range (distribution points with red color) of *Teucrium luteum* subsp. *gabesianum* (local endemic of Tunisia) occurring in Bordj Toual and Gabes [17], Kerkennah Islands [45], Sefnou, Gremdi and Roumadiya [47], El Ataya (this study), Grande Kuriate Island [46], Kerker and Mesjed-Aïssa [47], Matmatas and Gafsa [17,49], and Djerba Island [48].

The ecological profile of *T. luteum* subsp. *gabesianum* was created using distribution points taken from literature sources [17,45,46,47,48,49], personal herbarium records, and occurrences retrieved from the GBIF portal (https://www.gbif.org/occurrence/search?taxon_key=3895614 accessed on 13 December 2021) (Figure 1). Based on these records, we calculated minimum, maximum, and average temperatures of the distribution sites and mean values of 19 standard bioclimatic variables similar to [23], using historical climate data of 30 sec pixel size from the website WorldClim (https://www.worldclim.org/data/worldclim21.html, accessed on 13 December 2021). The ecological profile of *T. luteum* subsp. *gabesianum* was created with GIS, using the following layers:

(a) WorldClim version 2.1 with minimum, maximum, and average temperature (°C), as well as precipitation values (mm) and data for 19 bioclimatic variables for every month derived from 1970–2000 in raster resolution of 1 km^2^, and
(b) *Teucrium luteum* subsp. *gabesianum* distribution points raster files (Figure 1).

### 2.3. Seed Collection and Storage

**Figure 2:**
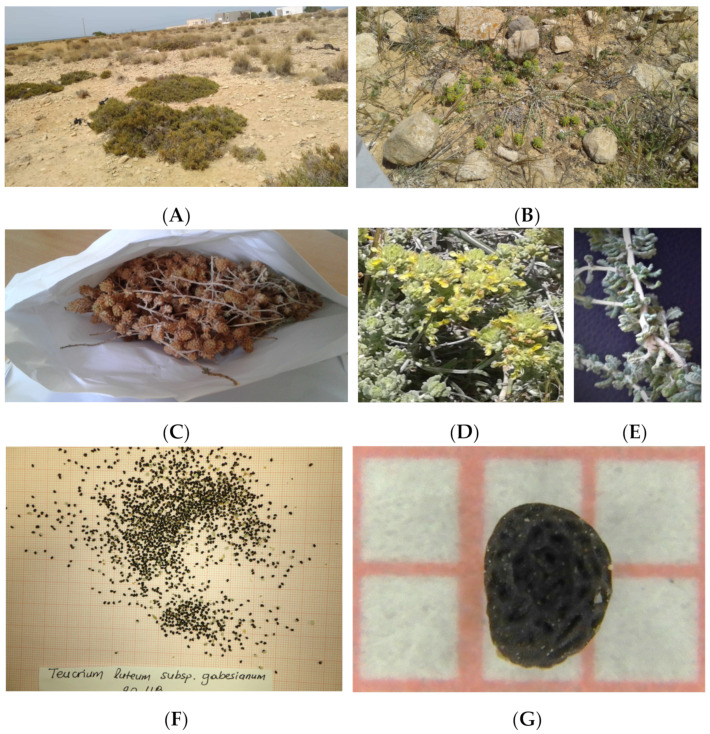
(**A**) Wild habitat; (**B**) Prostrate habit of a wild-growing individual; (**C**) Collected inflorescences; (**D**,**E**) Inflorescences and leaves; (**F**) Extracted seeds and morphology of a ripe individual seed (**G**) of *Teucrium luteum* subsp. *gabesianum*.

To obtain a snapshot of the local genetic diversity of *T. luteum* subsp. *gabesianum* of Île Kerkennah in Tunisia, dry inflorescences were collected by hand (12 June 2019) from 20 individual wild-growing plants in their natural habitat (Figure 2), at 1 m above sea level (34°49′46.9″ N, 11°14′50.2″ E). The plant material was collected after receiving special permission from the Ministry of Higher Education and Scientific Research of Tunisia (Ministère de l’Enseignement Supérieur & de la Recherche Scientifique, MESRS). After the limited harvesting of inflorescences form wild-growing individuals, plant material (inflorescences) was placed on filter papers at room temperature to dry naturally for 15 days. Seed extraction was conducted by hand-rubbing. Subsequently, seeds were separated from debris by sieving and flowing. The cleaned seeds were transferred at the seed bank of the Institute of Plant Breeding and Genetic Resources, Agricultural Organization Demeter in Thessaloniki, Greece, where they were taxonomically identified and assigned an IPEN (International Plant Exchange Network) accession number TN-1-BBGK-20,118 (Figure 2). The cleaned seeds remained in glass containers in the seed bank (3–5 °C) until further experimentation.

### 2.4. Germination Tests

Germination experiments were performed in December of 2019 at the facilities of the Laboratory of Floriculture, School of Agriculture, Aristotle University of Thessaloniki. The germination response of *T. luteum* subsp. *gabesianum* seeds to temperature was evaluated in growth chambers (CRW-500SD Chrisagis, Athens, Greece) at four constant temperatures (10, 15, 20, and 25 °C), with four sets of 20 seeds treated under each temperature regime. The seeds were placed on filter paper, moistened with distilled water in 9-cm sterile plastic Petri dishes. The Petri dishes were randomly arranged on the growth chambers shelves under a 12-h light/12-dark photoperiod. Filter paper was kept moist by adding distilled water when needed throughout the experimental period. The number of germinated seeds was recorded every five days for a period of 45 days, and thereafter, they were removed from the petri dishes. The evidence for seed germination was radicle protrusion from the seed coat.

### 2.5. Molecular Markers and PCR Procedures

Genomic DNA was extracted from young leaves of cultivated seedlings of *T. luteum* subsp. *gabesianum* (±250 mg of fresh tissue) according to a modified CTAB protocol [50]. DNA was electrophoresed on a 1% agarose gel, and the concentration was estimated using a spectrophotometer (NanoDrop™ One, Thermo Fisher Scientific, Lexington, MA, USA). DNA templates were PCR amplified using oligonucleotide primers (VBC-BIOTECH GmbH, Wien, Austria) for the following molecular markers: *18S–26S* (417 bp), *mat*K (845 bp), *pet*B/*pet*D (1047 bp), *rbc*L (651 bp), *rpo*C1 (492 bp), *trn*H/*psb*A (460 bp), *trn*L/*trn*F (893 bp), and *psb*K/*psb*I (426 bp). Sequences of the oligonucleotide primers, PCR thermal profile, reagents, sequencing of PCR products and analysis of DNA sequences were conducted following the methodology described in previous studies [51]. New sequence information of *T. luteum* subsp. *gabesianum* was deposited in GenBank, obtaining specific accession numbers for each of the above molecular markers (OM292866-72 and OM310764). Pairwise genetic distance and a neighbor-joining tree were constructed using MEGA 11.0 (Molecular Evolutionary Genetics Analysis) software [52].

### 2.6. Pilot Cultivation of Seedlings, Transplantation, and Fertilization Treatments

The germinated seeds of *T. luteum* subsp. *gabesianum* from the previous experiments were placed in plastic pots (6 × 6 × 6.5 cm^3^ dimension) filled with a 3:1 (*v*/*v*) mixture of enriched peat (TS1, Klasmann-Deilmann, Geeste, Germany) and perlite (Isocon, Athens, Greece). The germinated seeds were carefully covered with sand and the pots were positioned on the greenhouse bench. Pots were watered regularly to ensure desired substrate moisture for seedling growth.

The seedlings of *T. luteum* subsp. *gabesianum* were grown in the above pots until the development of a good root system. Later on (mid-March), the seedlings were carefully transplanted into new pots (8.5 × 8.5 × 9.5 cm^3^ dimension) containing a mixture of soil, enriched peat (TS2, Klasmann-Deilmann, Geeste, Germany), and perlite (Isocon, Athens, Greece) at a 4:5:1 (*v*/*v*) ratio. The fertility of the soil used in the above substrate was checked prior to mixing, and a sample of approximately 1.5 kg soil was taken for chemical analyses. The results of chemical analyses are presented in Table 1.

After the seedlings were transplanted, the pots were randomly divided into three groups, with eight replicates each. Integrated nutrient management (INM) was applied in the seedlings of the first group, chemical fertilization (ChF) was applied in the second group, and the third group was used as control with no application of fertilizers. Both types of fertilizers were applied through foliar spray application. INM fertilization by foliar application consisted of nutrient solution with THEORUN at 7 mL/L, THEOCAL at 1.5 g/L, THEOFAST at 5 mL/L, 10-47-10 (AGRI.FE.M. LTD Fertilizers, Aspropirgos, Greece) at 3.2 g/L, K_2_SO_4_ (0-0-52, AGRI.FE.M. LTD Fertilizers, Greece) at 2.07 g/L, micronutrients (Plex Mix, AGRI.FE.M. LTD Fertilizers, Aspropirgos, Greece) at 1.5 mL/L, and MgSO_4_ (Mg 25.6%, AGRI.FE.M. LTD Fertilizers, Aspropirgos, Greece) at 0.6 g/L [13,14]. The conventional fertilization by foliar application used nutrient solution consisted of NH_4_NO_3_ (34,4-0-0, Neofert^®^, Neochim PLC, Dimitrovgrad, Bulgaria) at 2.7 g/L, Ca(NO_3_)_2_ (NITROCAL, Agrohimiki, Patras, Greece) at 1.7 g/L, 10-47-10 at 3.2 g/L, K_2_SO_4_ (0-0-52) at 2.27 g/L, micronutrients Plex Mix at 1.5 mL/L, and MgSO_4_ (Mg 25.6%) at 0.6 g/L [13,14]. Fertilizations started in mid-March and were completed in mid-June. The plants were grown inside a glasshouse of the laboratory of Floriculture, School of Agriculture within the farm campus of the Aristotle University of Thessaloniki. Plants were irrigated every three days during the experimental period.

### 2.7. Morphological and Physiological Measurements of Seedlings

The effect of fertilization treatment on several morphological traits was evaluated at the end of June. Measurements of the main shoot height (SH) and root collar diameter (RCD) were taken from all plants of each treatment, using a metal ruler and a digital caliper, respectively. The number and length of apex shoots (>3 cm) from each plant per treatment was also recorded. In addition, a random sample of five plants per treatment was taken to assess root dry biomass (RDB), and above ground part dry biomass (AGDB). The tissue nutrient content of the samples was also measured. The dry weight of plants was calculated after oven drying at 74 °C for 48 h.

At the end of the experiment, the photosynthetic rate (μmol m^−2^ s^−1^) was measured with a portable gas exchange system LCi ADC Gas Analyzer (ADC BioScientific Ltd., Hoddesdon, UK). A cluster of top leaves was clipped in a small cyclical leaf chamber with a diameter of 16.5 mm and exposed window area of 2.16 cm^2^. All measurements were taken on sunny days between 11:00 a.m. and 02:00 p.m. with ambient temperatures ranging from 25.1 °C to 29.8 °C and leaf chamber temperature from 24.8 to 31.3 °C, while the ambient CO_2_ concentration (Cref) was 322.18 µmol mol^−1^, and the chamber water vapor pressure was 6–8 m bar.

### 2.8. Plant Tissue Analyses of Seedlings

All dried parts (including shoots and leaves) of each treatment were ground through a 40-mesh sieve to determine the tissue nutrient concentration in *T. luteum* subsp. *gabesianum*. Three samples of fine powder were made corresponding to the three fertilization treatments (ChF, INM, control) applied. Each sample was further divided into three subsamples of ca. 0.25 g each, and each subsample was disorganized by the method of wet oxidation using a triple acid mixture of H_2_SO_4_, HNO_3_, and HClO_4_ in a ratio of 5:1:1 at 80 °C until a transparent solution was obtained [53]. The digested samples were filtered using Whatman No. 42 filter papers and the filtrates were finally complemented with distilled water up to 50 mL. The solutions were analyzed for total P colorimetrically according to the Molybdenum Blue Method by using a Shimadzu spectrophotometer model UV-1201V [54]. The total concentrations of magnesium (Mg), potassium (K), calcium (Ca), sodium (Na), copper (Cu), iron (Fe), zinc (Zn), and manganese (Mn) were determined by atomic absorption spectroscopy (Perkin-Elmer Analyst 300). Acetylene gas was used as fuel and air as a supportive agent. An oxidizing flame was used in all cases. Furthermore, for the determination of N, three sub-samples of ca. 0.25g each of the powder were taken from each sample. Total N was determined by the Kjeldahl method [55].

### 2.9. Statistical Analysis

We used a completely randomized design for the experiments. The data were subjected to analysis of variance (one-way ANOVA), and the comparisons of the means were performed with Duncan’s test at significance level *p* ≤ 0.05 [56]. Prior to the ANOVA, only the germination percentage data was transformed to arc-sine square root values [57].

## 3. Results

### 3.1. Overview of the Utilization Potential

**Figure 3:**
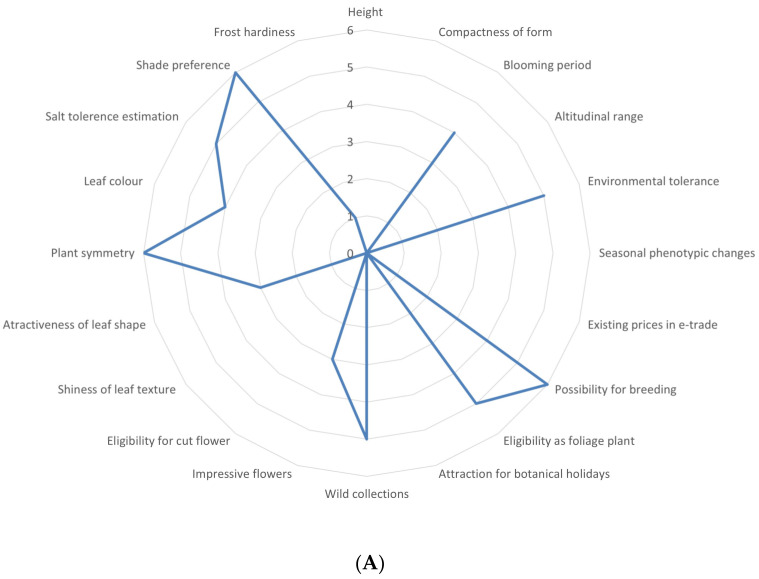

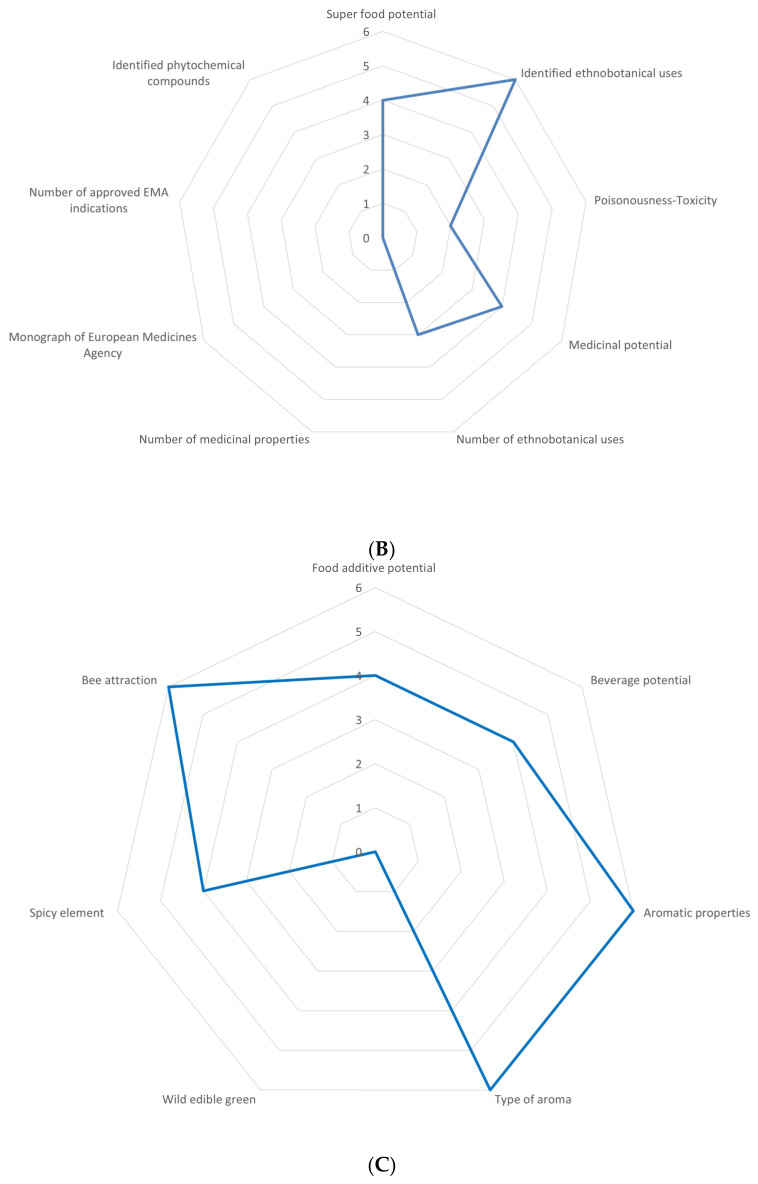
Multifaceted evaluation of *Teucrium luteum* subsp. *gabesianum* in different sectors of economy with obtained scores for: (**A**) 20 ornamental-horticultural attributes resulting in 44.16% of the optimum possible score [20]; (**B**) Nine medicinal-cosmetic attributes reaching 35.19% of the optimum possible score [18], and (**C**) Seven agro-alimentary attributes providing 71.43% of the maximum possible score [19].

*Teucrium luteum* subsp. *gabesianum* showed 44.16% of the optimum possible overall score in the ornamental-horticultural sector. This score ranked it among the top-three among 82 Tunisian local endemics (Figure 3A) with an interesting general potential (the top-evaluated Tunisian endemic species was *Limonium byzacium* Brullo & Erbern with 47.5%). In different subsectors of the ornamental-horticultural industry, *T. luteum* subsp. *gabesianum* was assessed with above-average to high potential as pot/patio plants with 56.25% of the optimum possible score, while it may be possibly eligible for landscaping and xeriscaping applications (40.86 and 39.59% of the possible optimum score, respectively) [20].

*T. luteum* subsp. *gabesianum* scored 35.19% in the medicinal-cosmetic sector (Figure 3B) and was included as seventh among the top 10 cases of 82 local Tunisian plants, mainly due to its associated ethnobotanical knowledge [18]. This taxon scored 71.43% in the agro-alimentary sector (Figure 3C), and it was included among the top-three cases among the 82 local Tunisian endemics examined [19], due to its aromatic properties, type of aroma, and bee attraction ability.

Nonetheless, examining how feasible the sustainable exploitation is for this taxon (Level II evaluation), *T. luteum* subsp. *gabesianum* received a low score (25% of the maximum possible) in comparison to other local endemic plants of Tunisia [20], ranking it very low (<35%). This assessment outlines important gaps related with its possible exploitation. In this fashion, the readiness timescale for value chain creation for *T. luteum* subsp. *gabesianum* (Level III evaluation) was estimated as achievable but with an indeterminable horizon [20].

### 3.2. Molecular Characterization and Annotation in GenBank

The evaluation of eight different molecular markers in *T. luteum* subsp. *gabesianum* and the DNA sequences obtained were analyzed and submitted to the GenBank database. In the absence of previously submitted DNA sequences in GenBank for *T. luteum* subsp. *gabesianum*, these data offer explicit genetic information allowing for DNA comparisons with other *Teucrium* species.

**Figure 4:**
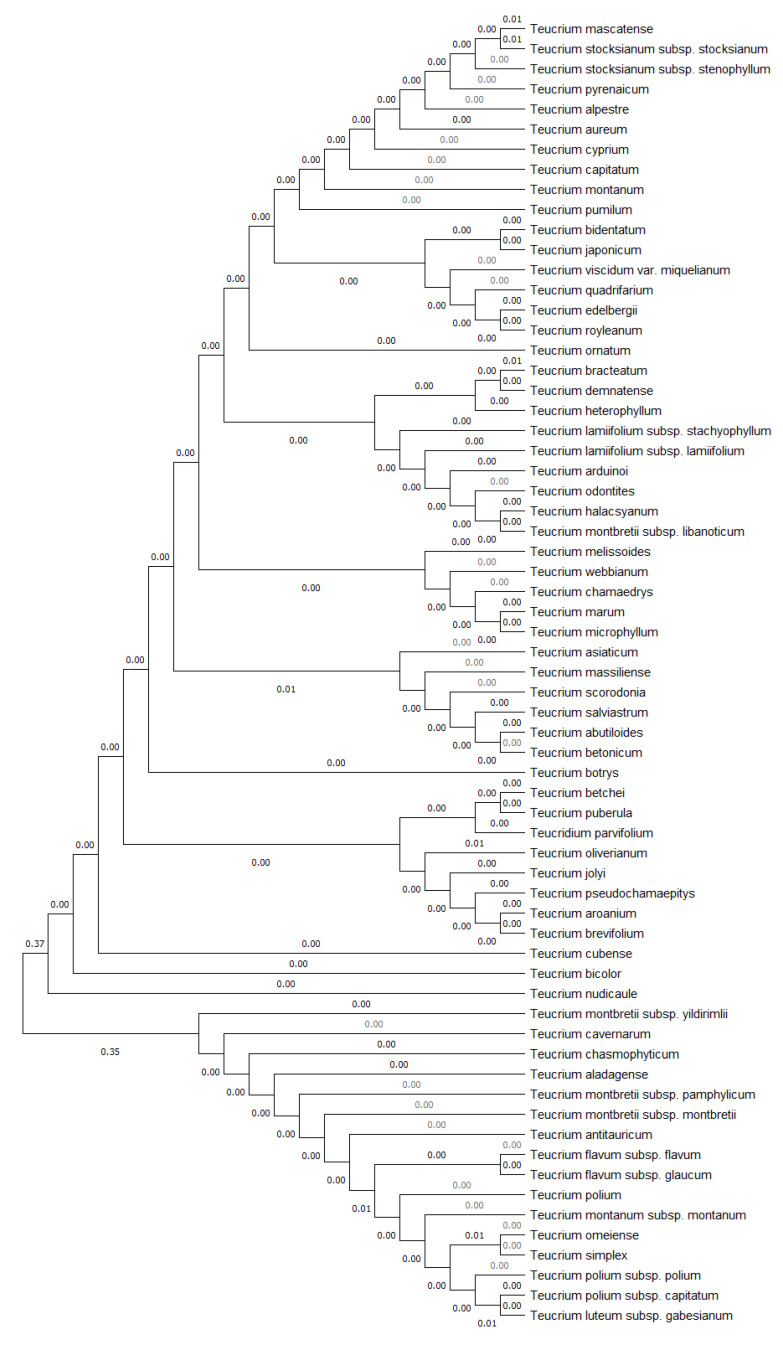
Phylogenetic tree of the genetic distances between *Teucrium luteum* subsp. *gabesianum* and 64 other *Teucrium* taxa (species and subspecies) retrieved from GenBank based on the *trn*L-*trn*F intergenic spacer sequences using the neighbor-joining method.

After annotating the DNA sequences of *T. luteum* subsp. *gabesianum* in GenBank, the genetic variation with respect to several *Teucrium* species was revealed (Table 2). The DNA sequence of *T. luteum* subsp. *gabesianum* for maturase (*mat*K) gene was matched with 35 *Teucrium* accessions, differing from 1 bp (*T. montanum* L., *T. nummularifolium* Baker, and *T. polium*) up to 19 bp (*T. betchei* F. Muell). The annotation of ribulose 1,5-bisphosphate carboxylase/oxygenase (*rbc*L) gene sequence of *T. luteum* subsp. *gabesianum* was matched with 23 *Teucrium* accessions, which differ from 4 bp (*T. mascatense, T. stocksianum* subsp. *stenophyllum*, and *T. stocksianum* subsp. *stocksianum*) to 12 bp (*T. flavum*). The locus of intergenic spacer between tRNA-His (*trn*H) and photosystem II protein D1 (*psb*A) gene of *T. luteum* subsp. *gabesianum* was matched with 25 *Teucrium* accessions, differing to 35 bp (*T. divaricatum* Sieber ex Heldr) up to 158 bp (one accession of *T. ornatum*). The external transcribed spacer between the *18S* and *26S* ribosomal RNA genes of *T. luteum* subsp. *gabesianum* was only matched to one accession of *T. ornatum*, showing a 102 bp difference (including gaps). The locus between the cytochrome b6 (*pet*B) and cytochrome b6/f complex subunit IV (*pet*D) genes (*pet*B/*pet*D molecular marker) of *T. luteum* subsp. *gabesianum* was matched with seven *Teucrium* accessions, which differ from 25 bp (*T. stocksianum* subsp. *stenophyllum* and *T. stocksianum* subsp. *stocksianum*) up to 51 bp (*T. mascatense*). The locus of intergenic spacer between the tRNA-Leu (*trn*L) and the tRNA-Phe (*trn*F) genes of *T. luteum* subsp. *gabesianum* was matched with 64 different *Teucrium* species; therefore, a phylogenetic dendrogram was constructed to identify the genetic distance among *Teucrium* species (Figure 4).

The base pair differences of *T. luteum* subsp. *gabesianum* compared to 37 *Teucrium* accessions for *trn*L/*trn*F molecular marker are presented in Table 2. The RNA polymerase beta’ subunit (*rpo*C1) gene DNA sequence of *T. luteum* subsp. *gabesianum* was matched with 12 *Teucrium* accessions, differing from 3 bp (*T. stocksianum* subsp. *stenophyllum* and *T. stocksianum* subsp. *stocksianum*) up to 7 bp (two accessions of *T. mascatense*). Finally, the chloroplast *psb*K-*psb*I intergenic region of *T. luteum* subsp. *gabesianum* was matched with 14 *Teucrium* accessions, which varied from 3 bp (*T. stocksianum* subsp. *stenophyllum* and *T. stocksianum* subsp. *stocksianum*) up to 76 bp in *T. junceum* (A. Cunn. ex Walp) Kattari & Heubl.

**Figure 5:**
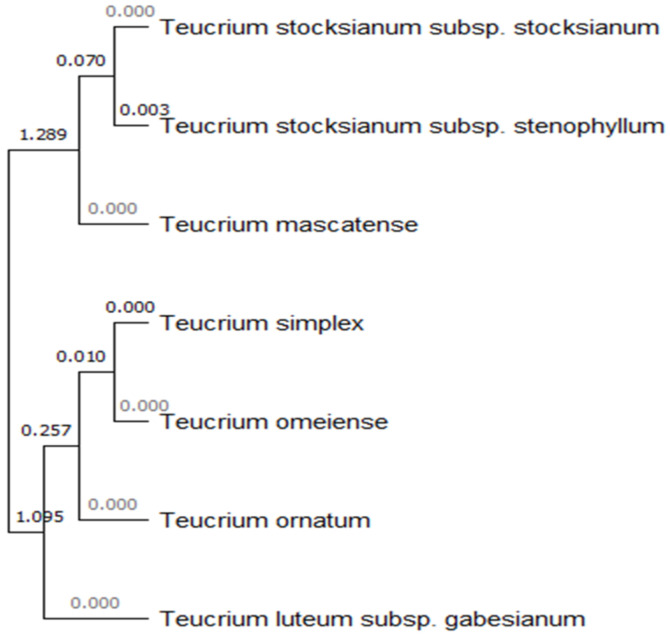
Phylogenetic tree of the genetic distances between *Teucrium luteum* subsp. *gabesianum* and six other *Teucrium* taxa (species and subspecies) with the complete chloroplast DNA genome retrieved from GenBank based on seven molecular marker sequences (*mat*K, *pet*D/*pet*B, *psb*K/*psb*I, *rbc*L, *rpo*C1, *trn*H/*psb*A and *trn*L/*trn*F) and using the neighbor-joining method.

The complete chloroplast DNA sequence is available in GenBank for six *Teucrium* species, i.e., *T. ornatum, T. simplex, T. omeiense, T. stocksianum* subsp. *stocksianum* and subsp. *stenophyllum*, and *T. mascatnese*. These data were used to construct a phylogenetic tree, presenting the genetic distances among species, based on seven molecular markers, i.e., *mat*K (845 bp), *pet*B/*pet*D (1047 bp), *rbc*L (651 bp), *rpo*C1 (492 bp), *trn*H/*psb*A (460 bp, *trn*L/*trn*F (893 bp), and *psb*K/*psb*I (426 bp) (Figure 5).

### 3.3. Ecological Profiling

**Figure 6:**
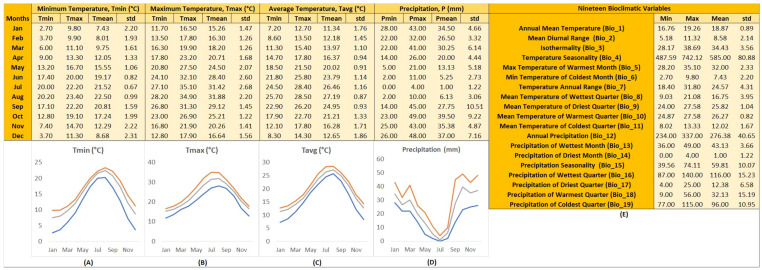
Ecological profile across the natural distribution range of *Teucrium luteum* subsp. *gabesianum* wild-growing populations in Tunisia linked in GIS with geodatabases (WorldClim version 2.1), providing values for: (**A**) minimum temperatures per month (°C), (**B**) maximum temperatures per month (°C), (**C**) average temperatures per month (°C), (**D**) precipitation per month (mm), and (**E**) calculated values for 19 bioclimatic variables. For (**A**–**E**) minimum, maximum, average, and standard deviation is shown based on data from 1970–2000. The colors of the plotted lines illustrate the minimum (blue), maximum (orange), and mean (grey) monthly values for temperature (°C) and precipitation (mm).

Using the natural occurrence records of *T. luteum* subsp. *gabesianum*, the respective GIS ecological profile was created to depict comprehensively the climatic conditions (temperature and precipitation) in which they naturally thrive on their wild-growing sites (Figure 6).

According to historical climate data about temperature-related attributes (Figure 6), the lowest value of average temperature was recorded in January (11.34 ± 1.76 °C) and February (12.18 ± 1.45 °C). From March (13.97 ± 1.10 °C) to June (23.79 ± 1.14 °C), the average temperature was evidenced to start rising in a stable rate, reaching a peak in July (26.46 ± 1.16) and August (27.19 ± 0.87 °C). After the summer season, the average temperature was shown to gradually decrease from 24.95 ± 0.93 °C in September to 12.65 ± 1.86 °C in December, when the coldest temperature was noted historically. The ecological profile of *T. luteum* subsp. *gabesianum* indicated that the minima of mean temperatures can reach 7.43 ± 2.20 °C in January and maxima of mean temperatures of 31.88 ± 2.20 °C in August. The mean diurnal range recorded was 8.58 ± 2.14 °C, and the annual mean temperature was 18.87 ± 0.89 °C. The relatively low values of these two bioclimatic variables, combined with the non-extreme temperatures across seasons (T_min_ of T_min_ = 2.70 °C in January, T_max_ of T_max_ = 35.10 °C in July) may indicate that *T. luteum* subsp. *gabesianum* wild-growing populations thrive in rather favorable environmental conditions for plant growth. In addition, the high temperature of T_min_ of T_min_ suggests that the winter season in the area of study is relatively mild.

In terms of precipitation-related attributes, the historical precipitation data in the areas where wild-growing populations of *T. luteum* subsp. *gabesianum* are found indicate that this taxon is able to withstand a dry environment, where the highest rainfall may be recorded in December, with a relatively low precipitation of 37.00 ± 7.16 mm. Similar rainfall was shown to occur in January (34.50 ± 4.66 mm), and after this period, precipitation was evidenced to start decreasing until May, a period with the lowest recorded mean value (13.13 ± 5.18 mm). Summer is the driest season in these areas, with only 1.00 ± 1.22 mm precipitation in July. After this period, precipitation historically starts to increase gradually from 27.75 ± 10.51 mm in September to 35.38 ± 4.87 mm in November (Figure 6).

### 3.4. Seed Germination Tests

**Figure 7:**
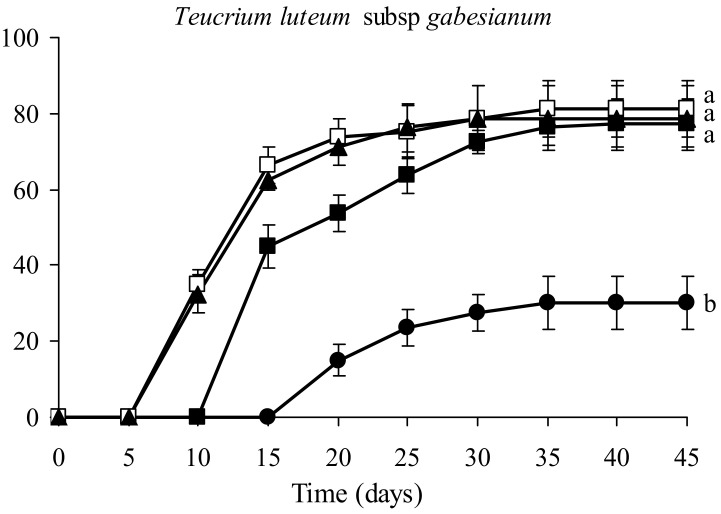
Cumulative seed germination percentage diagrams of *Teucrium luteum* subsp. *gabesianum* seeds incubated at different temperatures (● 10 °C, ■ 15 °C, □ 20 °C, and ▲ 25 °C). After Duncan’s test, means with different letters are statistically different at *p* < 0.05.

**Figure 8:**
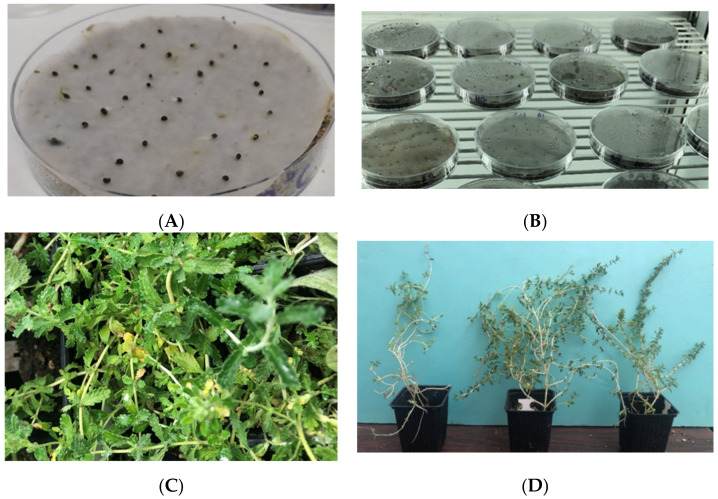
(**A**) Germinated seeds of *Teucrium luteum* subsp. *gabesianum* in petri dishes at 20 °C; (**B**) Petri dishes with *T. luteum* subsp. *gabesianum* seeds in a growth chamber at 20 °C; (**C**) Morphology of leaves of *T. luteum* subsp. *gabesianum* plants; (**D**) Plants of *T. luteum* subsp. *gabesianum* at the end of the fertilization experiment: control plant (left), plant with chemical fertilizers (ChF) applied (mιddle), and plant with integrated nutrient management fertilizers (INM) applied (right).

The germination percentages of *T. luteum* subsp. *gabesianum* seeds were affected significantly by temperature (Figure 7 and Figure 8A,B), and its seeds exhibited the highest germination percentages within a broad range of temperatures (15 to 25 °C). More precisely, the germination percentages of seeds incubated at 15, 20, and 25 °C were 77.5, 81.25, and 78.75%, respectively. However, seed germination was faster at higher temperatures (20 and 25 °C). In seeds incubated at temperatures of 20 or 25 °C, germination was recorded on the 10th day after the initiation of the germination test, whereas germination started five days later for seeds incubated at 15 °C. Germination was completed on the 30th day for seeds incubated at 25 °C and five days later for those incubated at 15 or 20 °C. Seeds incubated at 10 °C showed the lowest germination percentage (30%). In this temperature, germinated seeds were noted on the 20th day, and germination was completed on the 30th day.

### 3.5. Seedling Growth in Pilot Cultivation

Seedlings of *T. luteum* subsp. *gabesianum* were positively affected by fertilization treatments regarding the main shoot height. Root collar diameter of plants was not affected by fertilizers (Table 3). More precisely, plants that were fertilized with INM exhibited the highest shoot height. No significant differences were found either in root dry biomass or in above-ground dry biomass. The number of apex shoots per plant was affected by fertilizers, and the highest number was recorded in the ChF treatment (Table 3). The lowest number of apex shoots was noted in the control treatment. The highest photosynthetic rate was recorded in ChF fertilized plants, followed by the treatment of the INM type fertilizer, while control plants showed the lowest photosynthetic rate (Figure 8C,D).

### 3.6. Macro- and Micronutrient Content

The analysis of macronutrient content of *T. luteum* subsp. *gabesianum* leaves showed an increased concentration of potassium (K) in plants treated with organic fertilizer application compared to the control treatment (unfertilized plants) (Table 4). Regarding calcium (Ca) concentration, the lowest value was recorded in *T. luteum* subsp. *gabesianum* plants fertilized with the inorganic fertilizer, while no significant difference was detected between unfertilized plants and those fertilized with the INM fertilizer. With regard to micronutrient content, plants of the ChF treatment showed higher concentrations of iron (Fe) and zinc (Zn) compared to the INM fertilization treatment (Table 5). However, the highest concentration of both microelements was noticed in plants of the control treatment. No significant differences were found among treatments for manganese (Mn) concentration.

## 4. Discussion

### 4.1. Molecular Authentication (DNA Barcoding)

Species identification within taxa of genus *Teucrium* is particularly difficult due to a lack of stable and reliable taxonomic characters, leading often to numerous taxonomic disagreements especially regarding the members of section *Polium* [58,59,60]. To address such problems, targeted studies based on morphological traits (inflorescence, calyx teeth, hairiness, leaves edge, etc.), cytological, phytochemical, and molecular characterizations have been conducted [45,58,59,60,61,62,63,64]. In this frame, DNA barcoding of *T. luteum* subsp. *gabesianum* was conducted herein for the first time using seven chloroplast DNA markers and one nuclear ribosomal DNA marker to identify the genetic variation between *Teucrium* taxa and to provide a reference genetic DNA profile in GenBank. To date, no other sequence annotations exist in GenBank regarding *T. luteum* subsp. *gabesianum*. The seven chloroplast markers used herein for the molecular characterization of *T. luteum* subsp. *gabesianum* were proved efficient as they matched with 29 different *Teucrium* species and their multiple accessions. The whole chloroplast DNA genome has been completed for six *Teucrium* species, i.e., *T. stocksianum* subsp. *stocksianum* (MH325133), *T. stocksianum* subsp. *stenophyllum* (MH325131)*, T. mascatnese* (NC044073) [42], *T. ornatum* (MN814864), *T. simplex* (MN814872), and *T. omeiense* (MN814871), thus providing valuable information for the genetic classification of *Teucrium* taxa. Based on the comparison of seven molecular markers with the complete chloroplast genomes of six *Teucrium* species, the focal taxon herein *T. luteum* subsp. *gabesianum* is genetically closer first to *T. ornatum, T. omeiense*, and *T. simplex*, and subsequently to *T. stocksianum* subsp. *stocksianum*, *T. stocksianum* subsp. *stenophyllum*, and *T. mascatnese* (Figure 4). The molecular marker that was mostly applied in *Teucrium* taxa was *trn*L/*trn*F, with 100 accessions from 64 different species and subspecies. Genetic differentiation of *T. flavum* [39], *T. scorodonia* [65] and subspecies of *T. polium* [40] was studied using the *trn*L/*trn*F chloroplastic molecular marker and the nuclear ribosomal ITS region. Molecular analysis of *T. luteum* subsp. *gabesianum* compared to 20 representative *Teucrium* species confirmed a very close genetic relatedness to the Chinese endemic taxa *T. simplex* and *T. omeiense* and the West Mediterranean *T. polium*, followed by the Euro-Mediterranean *T. montanum* (Figure 5). The *18*S/*26*S nuclear ribosomal DNA molecular marker was matched only to one species of *T. ornatum* with 76% similarity. This is the first study of molecular characterization of *T. luteum* subsp. *gabesianum*, with additional registration of its DNA sequences in GenBank, aiming to contribute to future genetic diversity studies of the genus *Teucrium*. The findings reported herein also reflect the assumed taxonomic affinity of *T. luteum* subsp. *gabesianum* with other members of the Section *Polium*, as well as its former taxonomic placement as a subspecies of *T. polium* [17]. All target genes/genomic areas aforementioned are largely conserved in all plants; therefore, these markers may be used to outline single nucleotide polymorphisms (SNPs) indicating genetic fingerprints of particular *T. luteum* subsp. *gabesianum* populations, and they may also provide precise evidence for genetic circumscription of new *Teucrium* species. In addition, the molecular authentication of *T. luteum* subps. *gabesianum* permits traceability in traditionally used products that are sourced from the wild, as well as in future marketed products containing parts thereof.

### 4.2. Seed Germination Facilitated with GIS

Seeds of *T. luteum* subsp. *gabesianum* showed increased germination percentages without pre-treatments. In contrast, seeds of other species of the genus *Teucrium* (e.g., *T. marum* L. and *T. polium*) have dormant seeds right after collection, and specific treatments are needed for breaking seed dormancy [66,67]. However, in the present study, it was found that seed germination varied along a temperature gradient and was observed to have a specific temperature requirement. In *T. luteum* subsp. *gabesianum* seeds, the highest germination rate was observed in a range of constant temperatures from 15–25 °C. Seeds of *T. luteum* subsp. *gabesianum* mature and disperse in mid-summer. According to GIS-derived ecological profile, the autumn temperatures are favorable for the initiation of seed germination in the wild habitats of species. Furthermore, the increased precipitation over the autumn months creates the ideal conditions for seed germination. At the same time, the relatively mild winter ensure the growth of the emerging *T. luteum* subsp. *gabesianum* seedlings. Thus, it could be concluded that *T. luteum* subsp. *gabesianum* is well-adapted to arid and warm climate conditions. According to [66], the maximum germination rate of *T. marum* seeds is obtained at a temperature range of 15–20 °C. Furthermore, this temperature range has been discussed as ideal for seed germination in several species of the Mediterranean region [68,69]. The study of the germination capacities of nine Tunisian native species of the genus *Teucrium* at temperatures of 16 and 22 °C revealed a massive and rapid germination with above 60% for *T. compactum* Clement ex Lag., *T. alopecurus* de Noé and *T. spinosum* L., and lower germination percentage not exceeding 46% for *T. chamaedrys* L., *T. ramosissimum* Desf., *T. shoenenbergeri* Nabli, *T. fruticans* L., and *T. pseudochamaepitys* L. [70]. In general, it is considered that temperature is a key environmental factor responsible for the control of germination, and many species have a definite temperature range for effective seed germination [71], implying that seeds will germinate only under favorable environmental conditions for successful seedling establishment and survival. Furthermore, the rapid seed germination may be perceived as an adaptation mechanism avoiding dry unfavorable conditions for seedlings, which ensures the rapid establishment of seedlings in the period where the soil is moist.

### 4.3. Seedling Growth and Fertilization Response

It has been suggested that fertilization can help in maintaining an increased photosynthetic rate in plant leaves [72]. This was indeed confirmed by the findings of the present study as the photosynthetic rate of *T. luteum* subsp. *gabesianum* was positively affected and increased when chemical fertilization was used. In the same line, integrated nutrient management also increased its photosynthetic rate in comparison to control plants. Similar findings have been reported in another study on the endemic species of Tunisia *Marrubium aschersonii* Magnus where two different types of fertilizers were applied [44], showing that the use of fertilizers also increased the rate of photosynthesis as reported herein for *T. luteum* subsp. *gabesianum*. It has been reported that the use of nitrogen (N) can increase the potential activity and photochemical efficiency of PSII but can also reduce qN, thereby improving the photosynthetic performance of plants [73]. However, only coherent application of N fertilizer can advance the photosynthetic efficiency [74]. Similarly, P fertilizers favor nutrient synthesis, as well as transportation in plants, and thus they may advance plant growth, dry matter accumulation, and light use efficiency [75]. In general, fertilizer application enhances photosynthesis and the subsequent production in cultivated plants [76,77].

### 4.4. Re-Evaluation of Feasibility and Readiness Timescale for Value Chain Creation

The knowledge and data produced in the present study for *T. luteum* subsp. *gabesianum* may actually bridge extant applied research gaps, thus permitting the re-evaluation of *T. luteum* subsp. *gabesianum* in terms of feasibility for value chain creation and readiness timescale for sustainable exploitation in the future (Level II evaluation after [20]). When re-evaluation is attempted for the attributes scored in a previous investigation [20], the feasibility for value chain creation for *T. luteum* subsp. *gabesianum* is considerably improved from 25% to 51.39%, thus upgrading this taxon from <35% (very low class) to >50–55% (average). The difference in the Level II re-evaluation has been generated due to the higher *ex-situ* conservation availability due to score 3 (two stored accessions in two different institutions) compared to none (0 score); the seed propagation protocol developed herein, which corresponds to score 6 compared to an absence of data (score 0); the high germination rate reported herein (80%), equaling score 6 as compared to the absence of data (score 0); the species-specific cultivation needs revealed herein (score 6) compared to nothing known about cultivation (score 0); and the development of a coherent cultivation protocol (score 6) compared to the absence of data (0). After the informed re-evaluation for this taxon (Level III evaluation according to Krigas et al. [20], an upgrade is also outlined, upgrading this taxon from an ‘indeterminable’ readiness timescale as previously assessed in [20] to ‘achievable in the medium-term’ (class; >50–55%).

## 5. Conclusions

The study herein offered novel insight regarding the climate conditions that *T. luteum* subsp. *gabesianum* is naturally adapted to in its wild habitat. The detailed ecological profiling generated with GIS also informed the conditions possibly needed during *ex-situ* cultivation and acclimatization in the anthropogenic environment, such as ex situ conservation facilities. This profiling was predominantly useful to comprehend the effect of temperature on its seed germination and can be further used to produce detailed species-specific cultivation guidelines [21,22,23,44]. Moreover, successful seed propagation of *T. luteum* subsp. *gabesianum*, as first reported herein, can be considered the baseline for in situ and ex situ conservation efforts or targeted sustainable exploitation strategies specialized for this unique NUP of Tunisia with medicinal and ornamental potential [18,19,20]. The molecular authentication (DNA barcoding) of *T. luteum* subsp. *gabesianum*, which is also firstly reported herein may enable product traceability and future product design, offering, at the same time, a genetic reference for this taxon with regard to different *Teucrium* taxa. The first-time detected effects of chemical and integrated nutrient management fertilizers on the growth and pilot cultivation of *T. luteum* subsp. *gabesianum* seedlings taken together with the aforementioned results may help to pave the way for specific conservation efforts facilitating, at the same time, the sustainable exploitation of this local endemic plant of Tunisia with promising potential in different economic sectors. Coordinated efforts, legislative protection, and more targeted research (e.g., vegetative propagation trials, trial farm cultivations, agro-processing aspects, etc.) are certainly needed before stakeholder attention is attracted for the creation and establishment of an operative value chain for this highly promising species.

## Figures and Tables

**Table 1:** Chemical and physical properties of the soil used in pilot cultivation of *Teucrium luteum* subsp. *gabesianum* seedlings.

pH	Organic Matter (%)	Soluble Salts (mS/cm)	CaCO_3_ (%)	Mechanical Composition		
				Sand (%)	Silt (%)	Clay (%)
8.12	0.36	0.35	5.50	56.00	28.00	16.00
Concentration of macronutrient (ppm)						
	N-No_3_	P	K	Mg	Ca	
	8.00	8.00	104.00	842.00	>2000	
Concentration of micronutrient (ppm)						
	Fe	Zn	Mn	Cu		
	4.7	2.00	7.06	0.77		

**Table 2:** DNA sequence analysis and contrast of *Teucrium luteum* subsp. *gabesianum* with other *Teucrium* species and subspecies based on eight molecular markers. Different records indicate the number of detected nucleotide differences (including gaps) compared to DNA sequences of *T. luteum* subsp. *gabesianum*. Multiple numbers in columns show variation in detected genetic differences between different accessions available in GenBank.

*Teucrium* Taxon	*mat*K	*rbc*L	*trn*H/*psb*A *	*18S-26S*	*pet*B/*pet*D	*trn*L/*trn*F	*rpo*C1	*psb*K/*psb*I
*T. betchei*	19	10	-	-	-	58	6	68
*T. bidentatum*	10	-	57	-	-	29	-	-
*T. brevifolium*	-	-	53	-	-	59	-	-
*T. canadense*	12	6, 6	77	-	-	-	4	-
*T. chamaedrys*	11, 15	10	37, 43, 43	-	-	54	-	-
*T. flavum*	-	12	-	-	-	55, 56, 58, 58	-	-
*T. fruticans*	-	9	-	-	-	-	-	-
*T. heterophyllum*	-	5	-	-	-	21	-	-
*T. divaricatum*	10, 10	-	35	-	-	-	-	-
*T. junceum*	18	10	-	-	-	-	6	76
*T. japonicum*	-	-	52	-	-	26	-	30
*T. marum*	11	-	-	-	-	53, 54, 55, 55	-	-
*T. mascatense*	9, 9, 3	4, 4	-	-	51, 51	51, 51	7, 7	75, 75
*T. montanum*	2,1	-	42, 69	-	-	7, 18, 8	-	-
*T. nummularifolium*	1	-	-	-	-	-	-	-
*T. omeiense*	12	7	95	-	42	34	4	28
*T. ornatum*	15, 12, 11, 13	6, 6, 6	158, 54	102	41	36, 32	4	26
*T. parvifolium*	13	8, 9	-	-	-	35	5	31
*T. polium*	2, 1	-	51, 47	-	-	6, 5, 9, 11	-	-
*T. quadrifarium*	12	-	59	-	-	25, 27	-	-
*T. racemosum*	20	10	39	-	-	-	5	30
*T. scordium*	12, 12	-	49	-	-	-	-	-
*T. scorodonia*	11, 13	10, 10	-	-	-	40, 41, 42, 39	-	-
*T. simplex*	13	9	95	-	42	34	4	28
*T. stocksianum* subsp. *stenophyllum*	5, 3	4	51	-	25	38	3	3
*T. stocksianum* subsp. *stocksianum*	3	4	50	-	25	28	3	3
*T. tsinlingense*	12	-	-	-	-	-	-	-
*T. veronicoides*	-	-	70	-	-	-	-	28, 38
*T. viscidum*	9	6	77, 70, 72	-	-	27	-	29

* Analysis and alignment of the *trn*H-*psb*A molecular marker was completed for only 260 bp of *Teucrium luteum* subsp. *gabesianum*.

**Table 3:** Effect of integrated nutrient management fertilizers (INM) and chemical fertilizers (ChF) on characteristics related with growth and physiology of *Teucrium luteum* subsp. *gabesianum* seedlings. Means ± standard deviation values are shown. Values in the same row followed by the same letter are not significantly different (*p* > 0.05), according to Duncan’s test.

Characteristics	Control	INM	ChF
Shoot height (cm)	26.37 ± 3.92 ^b^	36.75 ± 4.03 ^a^	29.50 ± 5.96 ^b^
Root collar diameter (mm)	1.97 ± 0.30 ^a^	1.86 ± 0.37 ^a^	1.88 ± 0.19 ^a^
Number of apex shoots	6.37 ± 1.68 ^c^	12.62 ± 2.67 ^b^	16.87 ± 2.30 ^a^
Length of apex shoots (cm)	14.87 ± 2.29 ^ab^	16.50 ± 2.14 ^a^	12.75 ± 2.25 ^b^
Root dry biomass (gr)	0.20 ± 0.05 ^a^	0.18 ± 0.04 ^a^	0.14 ± 0.02 ^a^
Above ground dry biomass (gr)	0.75 ± 0.08 ^a^	0.82 ± 0.11 ^a^	0.80 ± 0.16 ^a^
Photosynthetic rate (μmol m^−2^ s^−1^)	1.92 ± 0.36 ^c^	3.80 ± 0.31 ^b^	4.75 ± 0.48 ^a^

**Table 4:** Macronutrient concentration of *Teucrium luteum* subsp. *gabesianum* young plants treated with inorganic and integrated nutrient management (INM) fertilizers (control: no fertilization).

Treatment	N (%)	P (mg/gr)	K (mg/gr)	Ca (mg/gr)	Mg (mg/gr)	Na (mg/gr)
Control	2.14 ^a^	2.10 ^a^	13.41 ^c^	8.37 ^a^	2.47 ^a^	4.47 ^b^
Inorganic fertilizer	2.07 ^a^	2.11 ^a^	15.39 ^b^	6.76 ^b^	2.62 ^a^	4.28 ^c^
INM fertilizers	2.42 ^a^	2.28 ^a^	16.48 ^a^	7.82 ^a^	2.44 ^a^	4.73 ^a^

Within the same column, the means are statistically different at *p* < 0.05 when they do not share a common letter. The comparisons were made using the Duncan’s test.

**Table 5:** Micronutrient concentration of *Teucrium luteum* subsp. *gabesianum* young plants treated with inorganic and integrated nutrient management fertilizers (control: no fertilization).

Treatment	Fe (ppm)	Mn (ppm)	Zn (ppm)	Cu (ppm)
Control	453.70 ^a^	60.95 ^a^	32.09 ^a^	4.63 ^b^
Inorganic fertilizer	380.49 ^b^	53.81 ^a^	27.51 ^b^	1.82 ^c^
Integrated nutrient management fertilizer	357.20 ^b^	62.43 ^a^	25.65 ^b^	88.01 ^a^

Within the same column, the means are statistically different at *p* < 0.05 when they do not share a common letter. The comparisons were made using the Duncan’s test.

## Data Availability

The data presented in this study are available on request from the corresponding authors.
